# Effects of acupuncture on brain tissue metabolism and neurological function in patients with ischemic stroke: a systematic review and meta-analysis

**DOI:** 10.3389/fneur.2026.1738752

**Published:** 2026-02-10

**Authors:** Song Li, Mengqi Yue, Xu Chen, Xiahui Zhang, Lijun Huang, Ziyi Wang, Yanling Jin, Qian Ma, Lei Ma, Jing Shi

**Affiliations:** 1 Heilongjiang University of Chinese Medicine, Harbin, China; 2 Yunnan Provincial Hospital of Traditional Chinese Medicine, The First Affiliated Hospital of Yunnan University of Chinese Medicine, Kunming, China; 3 Yunnan University of Chinese Medicine, Kunming, China

**Keywords:** acupuncture, brain tissue metabolism, ischemic stroke, magnetic resonance spectroscopy, meta-analysis

## Abstract

**Objective:**

This study aims to explore the effects of acupuncture on brain tissue metabolism and neurological function in regions centers of patients with ischemic stroke (IS).

**Methods:**

From the establishment of the database until May 20, 2025, a comprehensive search was conducted across several databases, including CNKI, WanFang, VIP Database, CBM, PubMed, Cochrane Library, Embase, and Web of Science. This search specifically targeted clinical randomized controlled trials (RCTs) that investigated the effects of acupuncture on cerebral tissue metabolism within the center of IS lesions and its subsequent impact on neurological function. The literature was meticulously screened, and information was extracted in accordance with the PRISMA guidelines. The quality of the literature was assessed using the risk of bias scale recommended in the Cochrane Handbook for Systematic Reviews of Interventions, version 5.1.0. Additionally, the quality of the included literature and the meta-analysis were evaluated using RevMan 5.4.

**Results:**

This study included 9 randomized controlled trials involving 602 patients. The meta-analysis results indicate that acupuncture treatment significantly improves the NAA/Cr ratio [MD = 0.19, 95% CI (0.14–0.24), *p* < 0.00001, 8 studies, 526 subjects] and reduces the Cho/Cr ratio [MD = −0.25, 95% CI (−0.36, −0.15), *p* < 0.00001]. However, no significant difference was observed in reducing the Lac/Cr ratio [MD = 0.04, 95% CI (−0.24, 0.32), *p* = 0.79]. Additionally, acupuncture treatment led to significant improvements in the NIHSS score [MD = −2.84, 95% CI (−3.76, −1.92), *p* < 0.00001], the FMA score [MD = 12.94, 95% CI (7.07, 18.81), *p* < 0.0001], and the MoCA score [MD = 3.20, 95% CI (2.30–4.10), *p* < 0.00001, 2 studies, 120 subjects] compared to non-acupuncture treatment. Overall, acupuncture demonstrated superior efficacy in improving the NAA/Cr, and Cho/Cr ratios, as well as the FMA, MoCA, and NIHSS scores, among IS patients.

**Conclusion:**

Adding acupuncture therapy to conventional treatment improves brain tissue metabolism and neurological function in patients with IS. It shows better efficacy compared to conventional treatment alone. However, evidence for specific outcome measures is limited. High-quality, large-scale RCTs are needed to strengthen the evidence base.

**Systematic review registration:**

https://www.crd.york.ac.uk/, CRD42024579263.

## 1 Introduction

Ischemic stroke (IS) is a disorder characterized by acute focal damage to the central nervous system due to the transient or permanent disruption of blood supply to the brain. It is currently one of the leading causes of adult mortality worldwide and a significant contributor to acquired disability and long-term functional impairment (1, 2). In China, although incidence and mortality rates show a declining trend, the burden of stroke has become a major public health issue due to its large population base and accelerating aging process (3). Following an IS, most patients experience varying degrees of neurological impairment. The effective treatment for IS involves promptly recanalizing occluded blood vessels while managing high-risk factors during the acute phase. The primary effective interventions include intravenous and/or arterial thrombolysis, which can restore neuronal function in the ischemic region and maintain white matter integrity in the affected area, thereby alleviating clinical symptoms associated with neurological dysfunction (4). However, these interventions are constrained by a limited time window and a heightened risk of bleeding. Consequently, many survivors are left with long-term neurological deficits that significantly impede their daily activities and overall functioning (5).

Currently, the primary therapeutic modalities for effectively treating neurological impairments following IS include physical therapy and pharmacological interventions. However, several significant challenges persist in long-term treatment, such as the potential for liver and kidney toxicity, gastrointestinal complications, high costs, and issues related to patient compliance (6–8). Consequently, there is an urgent need to explore alternative therapeutic methods that are simple, safe, cost-effective, low in side effects, and that promote high patient compliance through multi-target integration and modulation.

Acupuncture is a significant component of traditional Chinese medicine and has a long history of use in stroke treatment in China. This practice primarily aims to stimulate the body’s functions to achieve balance by inserting fine needles into specific acupoints, thereby facilitating recovery from the disease. The World Health Organization (WHO) recommends acupuncture as a complementary therapy for post-stroke rehabilitation (9). Evidence indicates that acupuncture has a pronounced therapeutic effect during the recovery phase of IS, notably in enhancing motor function, speech, cognitive abilities, and swallowing (10–12). The underlying mechanisms may be closely associated with the modulation of inflammatory responses, apoptosis, oxidative stress, cellular focal death, ferroptosis, and endogenous neurogenesis induced by acupuncture (13–17). Furthermore, research suggests that the therapeutic efficacy of acupuncture in IS patients is linked to brain tissue metabolism (18).

After ischemic injury, the body undergoes a series of complex changes, including neuronal excitotoxicity, energy metabolism imbalance, oxidative stress, inflammation, and apoptosis (19, 20). Moreover, IS is associated with metabolic disturbances. Nuclear magnetic resonance spectroscopy (MRS) can detect physiological and pathological changes in the energy metabolism of living tissues by using magnetic fields and radiofrequency pulses to noninvasively analyze specific nuclei and their compounds. Common indicators of energy metabolism include nitrogen-acetylaspartate (NAA), creatine (Cr), choline-containing compounds (choline, Cho), and lactic acid (Lac) (21). Several studies have confirmed that acupuncture can regulate brain tissue metabolism in patients with cerebral infarction, thereby improving motor, swallowing, and cognitive functions. However, there remains a lack of scientific evaluation regarding the effects of acupuncture on brain tissue metabolism and neurological function in patients with IS (18, 22, 23). Therefore, the aim of this study is to summarize and analyze data from randomized controlled trials, with a database established until May 20, 2025, focusing on the effects of acupuncture on brain tissue metabolism in patients with IS. By evaluating multiple outcome metrics, this study provides stronger evidence for clinical practice.

## 2 Materials and methods

### 2.1 Protocol and registration

The study protocol was registered with the International Prospective Register of Systematic Reviews (PROSPERO) (registration number CRD42024579263). Our research was conducted in strict adherence to the Preferred Reporting Items for Systematic Reviews and Meta-Analyses (PRISMA) reporting guidelines (24). The PRISMA checklist is available in Appendix S1.

### 2.2 Literature search

This study conducted a comprehensive search across eight databases, which included four English databases—PubMed, Cochrane Library, Embase, and Web of Science (WoS)—and four Chinese databases: the China National Knowledge Infrastructure (CNKI) Database, the Chinese Science and Technology Periodical (VIP) Database, the Wan Fang Database, and the Chinese Biological Medicine Database (CBM). The search period spanned from the establishment of each database until May 20, 2025, and included studies regardless of country, language, or publication status. The search utilized the terms ‘acupuncture,’ ‘ischemic stroke,’ ‘randomized controlled trial,’ and ‘magnetic resonance spectroscopies,’ along with a combined search of relevant free terms from the MeSH^1^ subject term search. The detailed search strategy is provided in Appendix S2.

### 2.3 Inclusion/exclusion criteria

The inclusion criteria for this study are as follows: (i) Study subjects: patients diagnosed with cerebral infarction; (ii) Study design: this study will encompass both domestic and international published randomized controlled trials (RCTs) that utilize magnetic resonance spectroscopy (MRS) to evaluate the effects of acupuncture treatment on brain tissue metabolism in patients with IS; (iii) Intervention: the acupuncture treatment will primarily consist of various forms, including traditional acupuncture, body acupuncture, head acupuncture, electroacupuncture, and warm acupuncture; (iv) Test group and control group settings: the test group will receive only acupuncture interventions, or acupuncture interventions in addition to those provided to the control group; patients in the control group will undergo non-acupuncture treatments, which may include conventional medication, rehabilitation therapy, and sham acupuncture; (v) Outcome measures: the study must report at least one cerebral metabolic index related to MRS, such as the NAA/Cr, Cho/Cr, or Lac/Cr ratios.

The exclusion criteria for this study comprised the following: (i) inability to obtain the full text; (ii) cross-sectional studies, case–control studies, and studies characterized by poor design and incomparable baseline characteristics; (iii) duplicate publications, retaining only the first instance of reported literature; (iv) reviews, meta-analyses, conference papers, dissertations, case reports, newspapers, and other non-original studies; and (v) studies that did not utilize NAA/Cr, Cho/Cr, or Lac/Cr ratios as indicators of outcome.

### 2.4 Literature screening and data extraction

This study involved two independent researchers who systematically screened the literature according to predefined inclusion and exclusion criteria. In instances of divergent findings, a third independent researcher was consulted to make a final judgment. Once consensus was reached among the three researchers on the literature for all included studies, basic information was extracted from these studies, including the first author’s name, publication date, randomization method, sample size, intervention details, intervention duration, and outcome indicators. For articles with incomplete data, the original authors were contacted via email or telephone to obtain any missing or unclear information.

### 2.5 Risk of bias and grading of evidence quality

This study was evaluated by two researchers using the risk of bias assessment tool recommended in the Cochrane Handbook of Systematic Reviews (version 5.1.0) for the included studies, with a third researcher designated to arbitrate in case of disagreement (25). The evaluation encompassed several domains, including randomized sequence generation, allocation concealment, blinding of both patients and investigators, blinding of outcome assessors, data completeness (including missed visits, dropouts, and non-compliance), selective reporting, and other potential bias factors such as sample size, baseline imbalance, and conflicts of interest. Each domain was categorized as having a low, unclear, or high risk of bias. Additionally, a meta-analysis was conducted using RevMan software (version 5.4).

This study evaluated the level of evidence for each outcome indicator. The quality of evidence was assessed using the Grading of Recommendations, Assessment, Development, and Evaluation (GRADE) system, which considers risk of bias, inconsistency, imprecision, indirectness, and publication bias. Evidence quality was categorized as high, moderate, low, or very low (26).

### 2.6 Data analysis

This study employed meta-analysis using RevMan 5.4 software. Mean differences (MD) were reported as effect sizes for continuous outcome indicators. For binary variables, relative risk (RR) and its 95% confidence interval (CI) were used to represent effect sizes. The *χ*^2^ test and *I*^2^ statistic were used to assess heterogeneity across studies. If *p* > 0.1 and *I*^2^ < 50%, we considered there was no significant heterogeneity among the included studies and used a fixed-effects model for the meta-analysis. Conversely, if *p* ≤ 0.1 or *I*^2^ ≥ 50%, indicating the presence of substantial heterogeneity, a random-effects model was employed instead. Additionally, subgroup analyses were conducted to examine sources of heterogeneity and enhance our understanding of the intervention effects. The funnel plot test was employed to evaluate publication bias.

## 3 Results

### 3.1 Study selection

**Figure 1:**
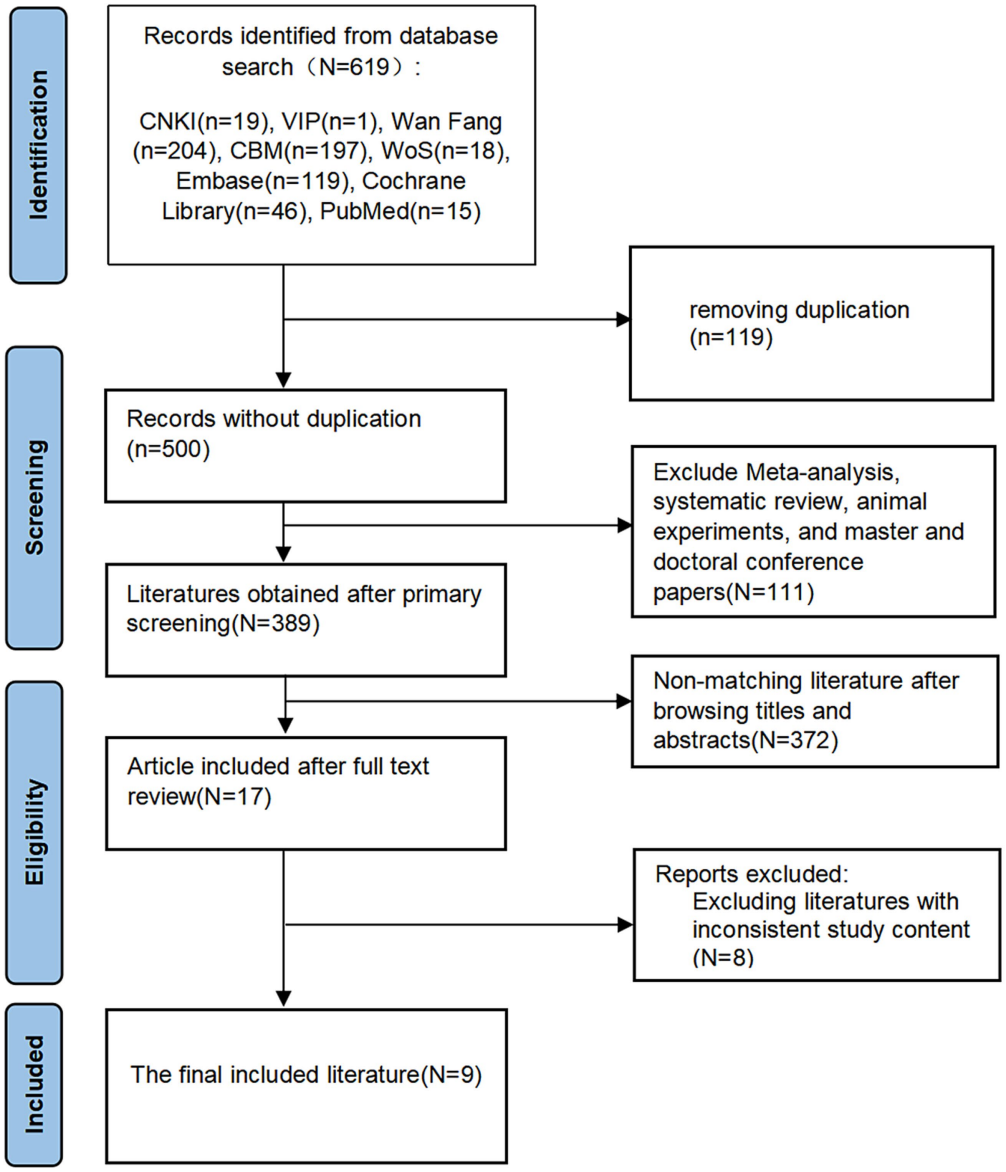
The details of the selection process.

This study searched eight Chinese and English databases using a defined search strategy, ultimately retrieving 619 documents. Our initial screening process excluded 119 duplicates, 99 conference papers and dissertations, and 12 reviews, systematic reviews, meta-analyses, and animal experiments. Upon reviewing titles, abstracts, and keywords, we identified 372 publications that did not align with the study’s content. A full-text review identified 8 articles inconsistent with the study’s objectives. Ultimately, nine articles were included in this study (18, 22, 23, 27–32) (Figure 1).

### 3.2 Study characteristics

This study comprises 9 RCTs involving 602 patients with cerebral infarction. The literature reviewed spans 2014 to 2020, and the treatment duration ranged from 15 days to 2 months. All patients received conventional therapy, while the experimental group additionally underwent acupuncture. Furthermore, two studies incorporated rehabilitation alongside the conventional treatment (18, 22). The control group interventions included conventional treatment (27–30), repetitive transcranial magnetic stimulation (rTMS) (32), and rehabilitation (18, 22, 23, 31). One study reported participant dislodgement or withdrawal (31). The primary outcome measures were brain tissue metabolism, neurological function, and cognitive function. These were assessed using the NAA/Cr ratio (18, 22, 27–32), Cho/Cr ratio (18, 22, 27–31), and Lac/Cr ratio for brain metabolism (23, 32), the National Institutes of Health Stroke Scale (NIHSS) score for neurological function, the Fugl–Meyer Assessment (FMA) score for limb motor function (22, 32), and the Montreal Cognitive Assessment (MoCA) score for cognitive function (22, 30). The characteristics of all nine studies are summarized in Table 1.

**Table 1:** Characteristics of included studies.

Study	Country	Age	Disease course (TG/CG)	Random method	Cases	Intervention time	Intervention		Outcome measures
		TG/CG			TG/CG		TG	CG	
Wang et al. (2014) (29)	China	56–80/58–81	–	Not mentioned	59/59	2 weeks	EA	CT	NIHSS, NAA/Cr, Cho/Cr
Wang et al. (2014) (30)	China	45–80	3–6 month	Not mentioned	30/30	12 weeks	MA	CT	MoCA, NAA/Cr, Cho/Cr
Zheng and Zheng (2013) (32)	China	51.4 ± 3.1	72 h	Random number table method	60/60	1 month	MA	rTMS	NIHSS, FMA, NAA/Cr, Lac/ Cr
Cai et al. (2020) (27)	China	51–80/49–79	14–23/15–23 days	Not mentioned	30/30	2 month	EA	CT	NIHSS, NAA/Cr, Cho/Cr
Lan et al. (2020) (23)	China	56.86 ± 11.25/57.96 ± 11.76	13.94 ± 8.67/14.47 ± 9.28 h	Not mentioned	38/38	4 weeks	EA	R	rNAA, Lac/Cr, sEMG
Zhang et al. (2020) (18)	China	70.10 ± 4.51/69.03 ± 4.70	24.63 ± 11.77/23.03 ± 7.47 days	Random number table method	30/30	6 weeks	MA + R	R	NAA/Cr, Cho/Cr, MoCA
Lin et al. (2018) (28)	China	56.3 ± 5.2	–	Random number table method	14/14	2 month	MA	CT	NAA/Cr, Cho/Cr
Zhang and Shen (2015) (31)	China	66 ± 11/63 ± 10	25.5 ± 7.3	Random number table method	30/30	8 weeks	EA	R	NAA/Cr, Cho/Cr
Jiang et al. (2016) (22)	China	55.80 ± 5.51/55.60 ± 8.61	91.90 ± 14.40/88.90 ± 15.60 days	Not mentioned	10/10	15 days	MA + R	R	FMA, MRS, NAA/Cr, Cho/Cr

TG, therapy group; CG, control group; MA, manual acupuncture; EA, electric acupuncture; CT, conventional therapy; rTMS, repetitive transcranial magnetic stimulation; R, rehabilitation; FMA, Fugl–Meyer Assessment; NIHSS, the National Institutes of Health Stroke Scale; MoCA, Montreal Cognitive Assessment.

### 3.3 Study design and risk of bias

**Figure 2:**
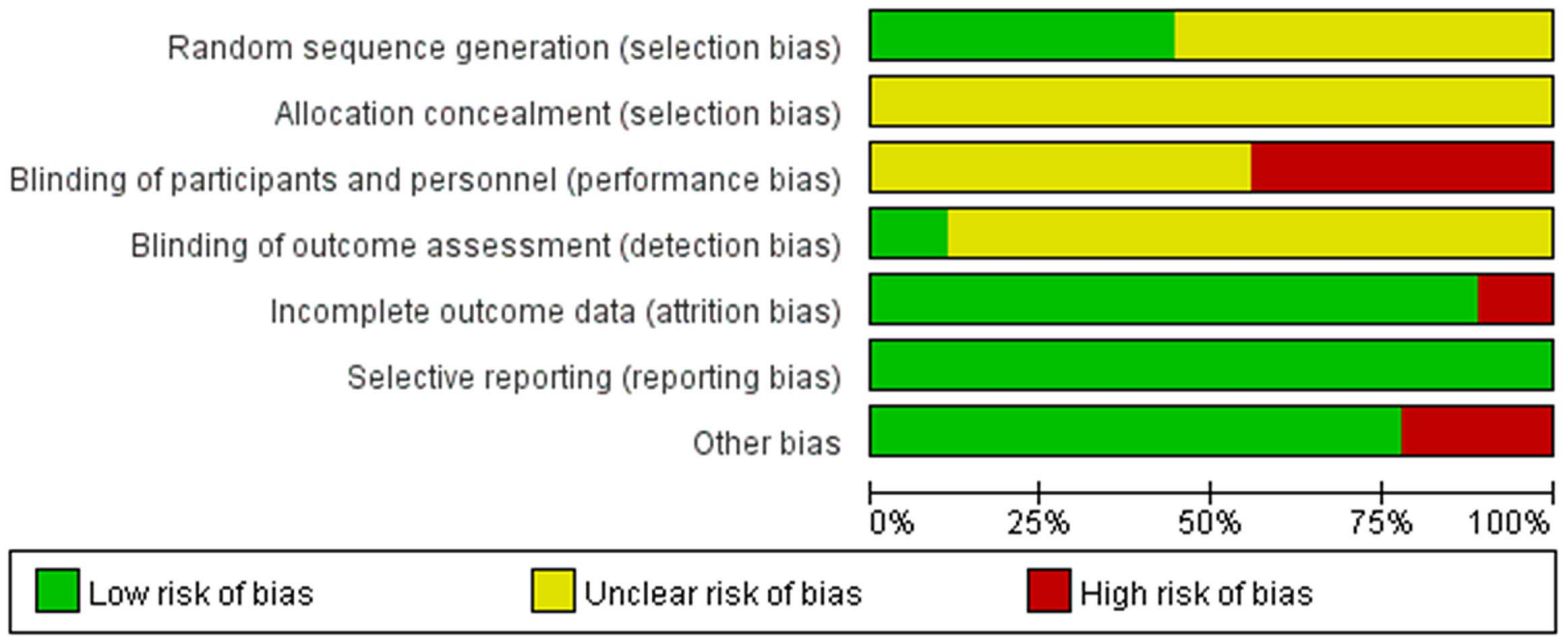
Risk of bias graph.

This study included nine investigations, all conducted in China and presented in Chinese. Four of the studies (18, 28, 31, 32) employed the random number table method, while the remaining five referred to random allocation but did not specify the method of randomization (22, 23, 27, 29, 30). Consequently, four studies were classified as having a ‘low risk’ of bias concerning the randomization method, and five studies were categorized as having ‘some concern’ regarding this aspect. The nine included studies (18, 22, 23, 27–32) did not mention the concealment of the allocation scheme, which raised ‘some concern’ about bias. Four studies (18, 22, 23, 30) indicated that the participants signed an informed consent form prior to participation, which was deemed ‘high risk’, whereas the remaining five studies did not address blinding (27–29, 31, 32), which was classified as ‘some concern’. One study (30) explicitly reported blinding of the assessment and measurement researchers, which was assessed as ‘low risk’, while the other eight studies (18, 22, 23, 27–29, 31, 32) did not mention this, leading to a classification of ‘some concern’. One study (31) reported participant disengagement and lost visits, categorizing it as ‘high risk’, while the other eight studies (18, 22, 23, 27–30, 32) provided complete data and were classified as ‘low risk’. None of the studies indicated selective reporting of results and were therefore considered to have a low risk. Additionally, two studies (22, 28) were classified as ‘high risk’ due to their small sample sizes (fewer than 30 cases). As illustrated in Figure 2. In the risk of bias plot, green indicates “Low risk of bias,” yellow indicates “Unclear risk of bias,” and red indicates “High risk of bias.”

### 3.4 Meta-analysis

#### 3.4.1 NAA/Cr ratio

**Figure 3:**
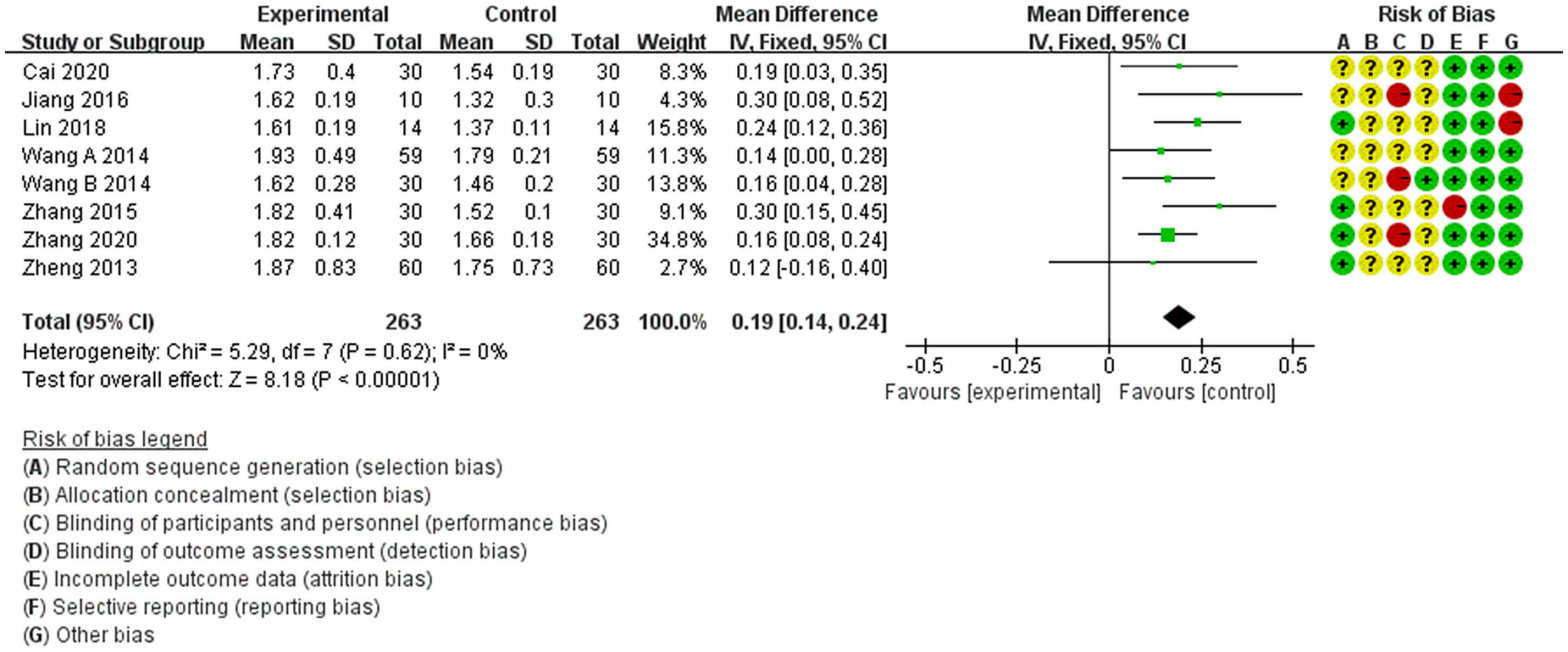
Forest plot of meta-analysis of the effect of acupuncture on brain tissue metabolism in IS patients, NAA/Cr ratio.

The study comprised nine separate investigations, of which eight (18, 22, 27–32) reported the NAA/Cr ratio as a specific outcome. A fixed-effects model was employed because there was no heterogeneity (*p* < 0.00001, *I*^2^ = 0%). The NAA/Cr ratio in the center of brain tissue lesions in patients with IS treated with acupuncture was found to be significantly superior to that of patients receiving non-acupuncture treatment [MD = 0.19, 95% CI (0.14–0.24), *p* < 0.00001, 8 studies, 526 subjects] (Figure 3). Furthermore, the results of the heterogeneity analysis indicated that the findings were reliable.

#### 3.4.2 Cho/Cr ratio

**Figure 4:**
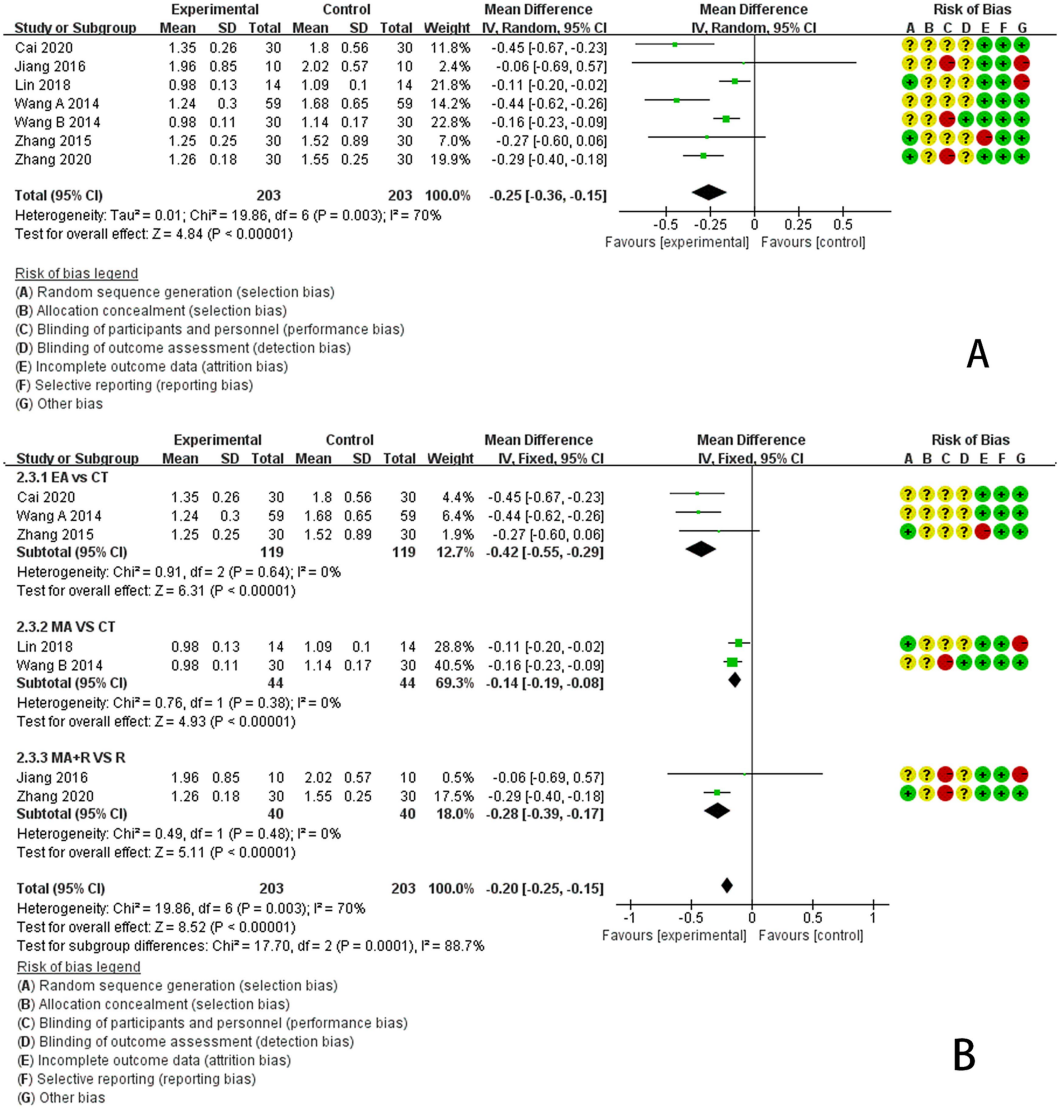
**(A)** Forest plot of meta-analysis of the effect of acupuncture on brain tissue metabolism in IS patients Cho/Cr ratio. **(B)** Subgroup analysis of the effect of acupuncture on brain tissue metabolism, Cho/Cr ratio in IS patients.

The Cho/Cr ratio was reported in seven studies, employing a random-effects model due to significant heterogeneity (*p* < 0.00001, *I*^2^ = 70%). The results indicated that acupuncture treatment was superior to the control group in reducing the Cho/Cr ratio in the center of brain tissue lesions among IS patients compared to non-acupuncture treatments [MD = −0.25, 95% CI (−0.36 to −0.15), *p* < 0.00001, 7 studies, 406 subjects]. However, the heterogeneity remained high (Figure 4A). Consequently, to identify the source of heterogeneity, a subgroup analysis was conducted to evaluate the effects of acupuncture on modulating the Cho/Cr ratio in the center of brain tissue foci, which serves as an outcome indicator for IS patients. The analysis was stratified by differences in acupuncture protocols across intervention groups to validate the reliability of the evidence supporting acupuncture’s superior efficacy in reducing brain lesion Cho levels compared with the control group. Results demonstrated that electroacupuncture treatment significantly outperformed rehabilitation therapy in reducing the Cho/Cr ratio at the center of cerebral lesion areas in IS patients [MD = −0.42, 95% CI (−0.55 to −0.29), *p* < 0.00001, 3 studies, 238 participants]. Manual acupuncture also outperformed rehabilitation therapy in reducing the Cho/Cr ratio at the lesion center in IS patients [MD = −0.14, 95% CI (−0.19 to −0.08), *p* < 0.00001, 2 studies, 88 participants]. Hand acupuncture combined with rehabilitation therapy also demonstrated superiority over rehabilitation therapy alone in reducing the Cho/Cr ratio at the lesion center in IS patients [MD = −0.28, 95% CI (−0.39 to −0.17), *p* < 0.00001, 2 studies, 80 participants], as illustrated in Figure 4B.

**Figure 5:**
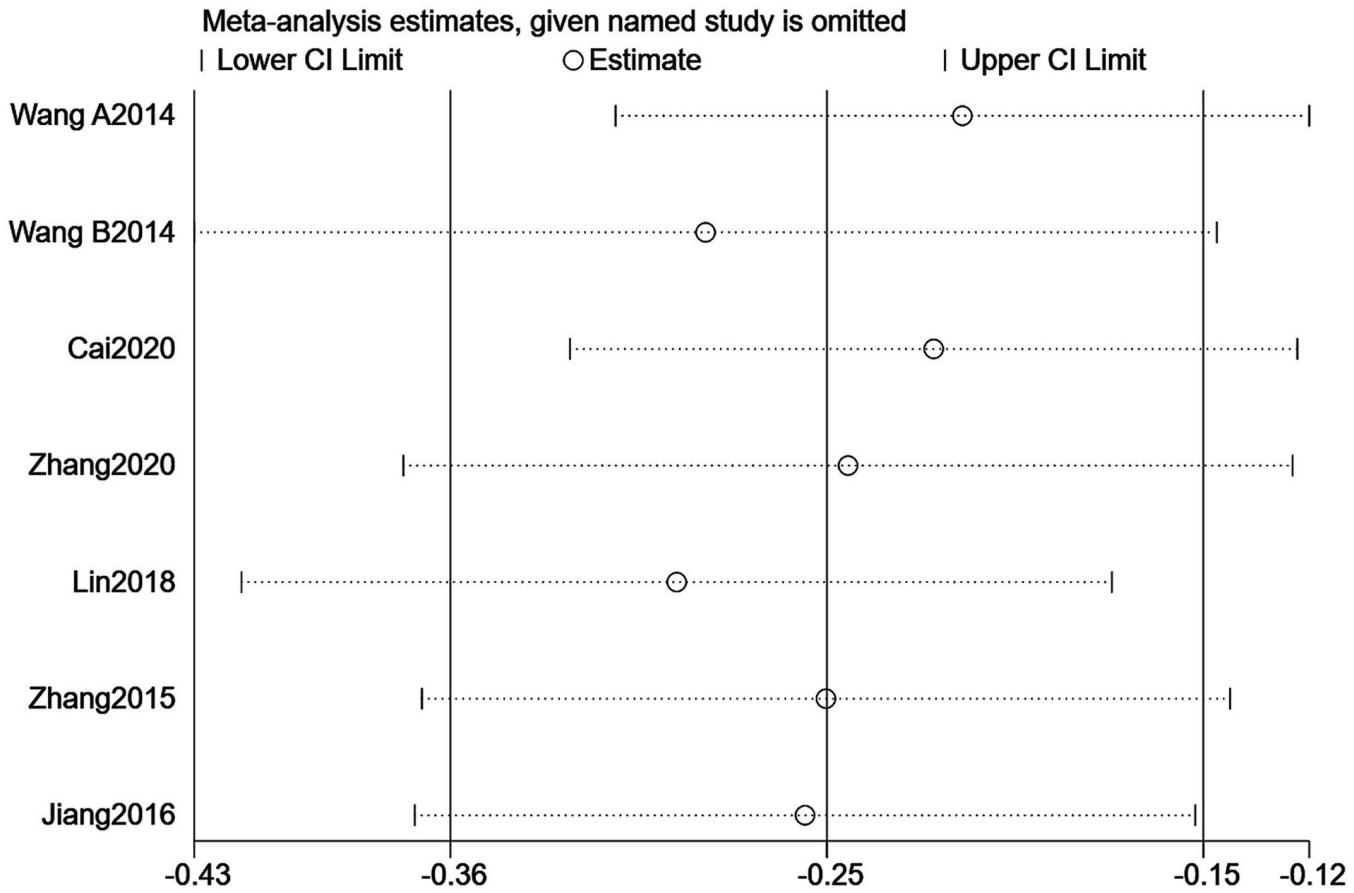
Sensitivity analysis plot for meta-analysis based on the Cho/Cr ratio.

To assess the stability of the meta-analysis’s pooled results, this study employed a stepwise exclusion sensitivity analysis. Results showed that after sequentially excluding the seven included studies, the pooled effect size of the remaining studies remained stable between −0.43 and −0.12. All 95% confidence intervals stayed within the null line, with no reversal in effect direction or change in statistical significance (Figure 5). This indicates that the pooled results of this meta-analysis demonstrate good stability and that the conclusions are highly reliable.

#### 3.4.3 Lac/Cr ratio

**Figure 6:**
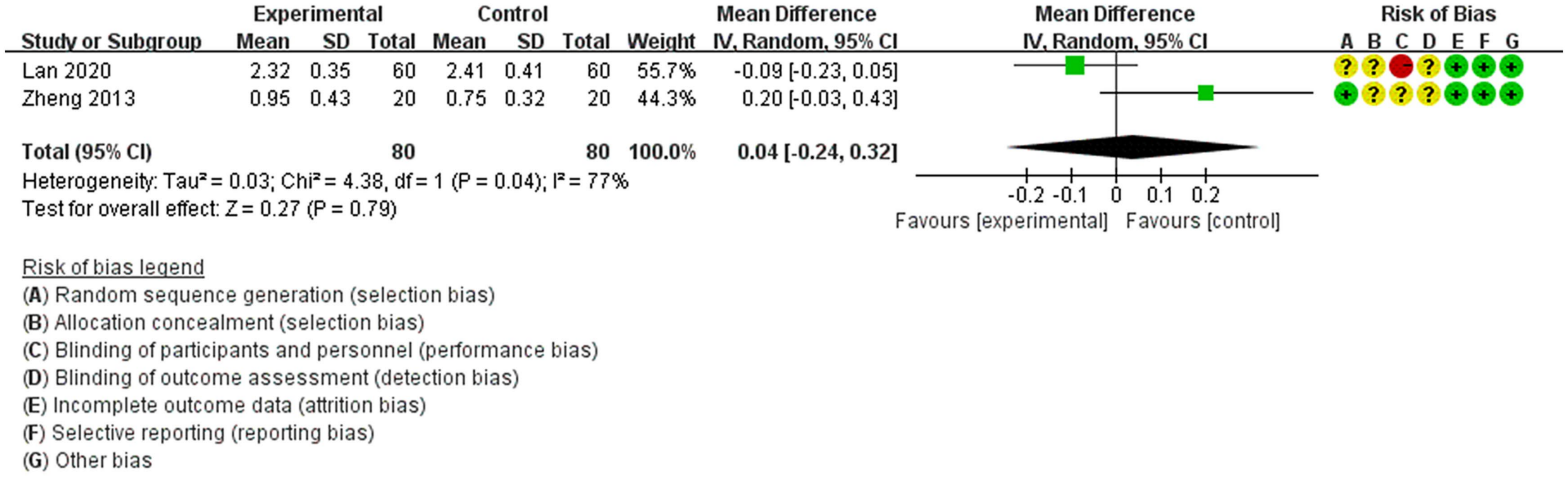
Forest plot of meta-analysis of the effect of acupuncture on brain tissue metabolism in IS patients, Lac/Cr ratio.

The two studies (23, 32) reported the Lac/Cr ratio as an outcome. A random-effects model was employed due to the observed heterogeneity (*p* < 0.79, *I*^2^ = 77%). Acupuncture treatment showed no statistically significant difference compared to non-acupuncture treatment in reducing lactate levels in the core lesion area of brain tissue in IS patients [MD = 0.04, 95% CI (−0.24, 0.32), *p* = 0.79, 2 studies, 160 subjects] (Figure 6). Additionally, the heterogeneity analysis indicated that the findings of these studies may be unreliable.

#### 3.4.4 NIHSS score

**Figure 7:**
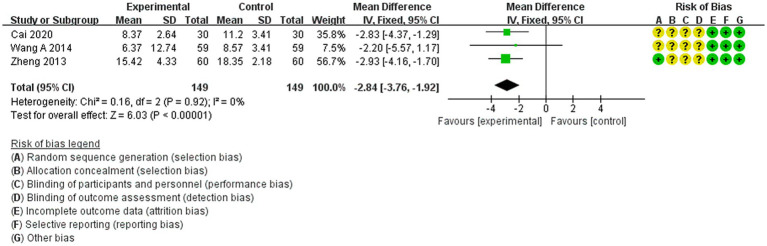
Forest plot of the meta-analysis of acupuncture on the NIHSS scores in IS patients.

The NIHSS scores were reported as outcomes in three studies (27, 29, 32). A fixed-effects model was employed because there was no heterogeneity (*p* < 0.00001, *I*^2^ = 0%). Acupuncture treatment demonstrated a significant improvement over non-acupuncture treatment in enhancing neurological function among patients with IS [MD = −2.84, 95% CI (−3.76 to −1.92), *p* < 0.0001, 3 studies, 298 subjects]. Furthermore, the heterogeneity analysis confirmed the reliability of these findings (Figure 7).

#### 3.4.5 FMA score

**Figure 8:**
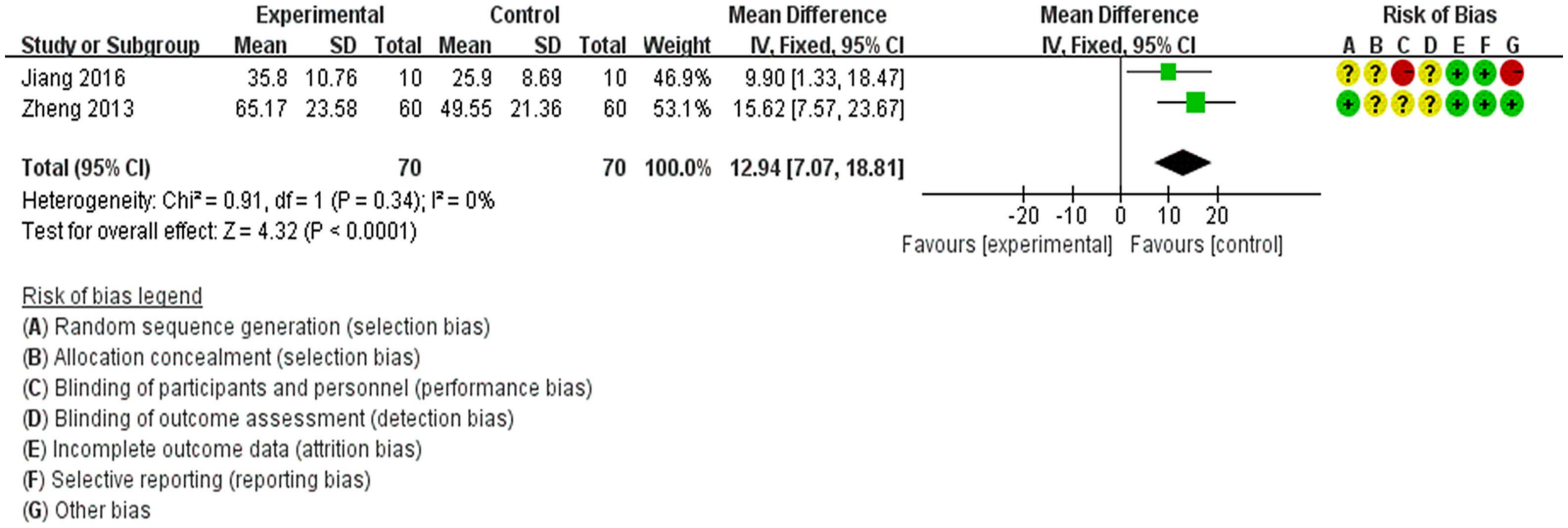
Forest plot of the meta-analysis of acupuncture on the FMA score in IS patients.

The FMA scores were reported as outcomes in two studies (22, 32). A fixed-effects model was employed because there was no heterogeneity (*p* < 0.00001, *I*^2^ = 0%). Acupuncture treatment demonstrated a significant improvement over non-acupuncture treatment in enhancing limb motor function among patients with IS [MD = 12.94, 95% CI (7.07–18.81), *p* < 0.0001, two studies, 140 subjects]. Additionally, the heterogeneity analysis indicated that the study findings were reliable (Figure 8).

#### 3.4.6 MoCA score

**Figure 9:**
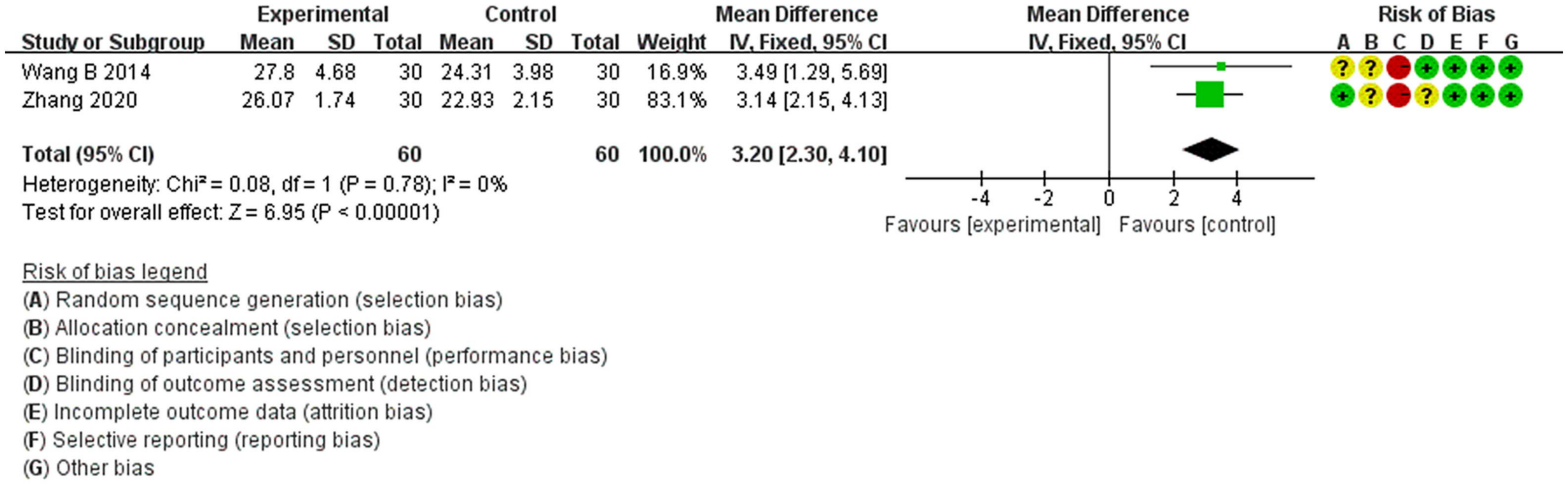
Forest plot of the meta-analysis of acupuncture on the MoCA score in IS patients.

This study encompassed nine distinct investigations, two of which (18, 30) specifically reported MoCA scores as outcomes. A fixed-effects model was employed because there was no heterogeneity (*p* < 0.00001, *I*^2^ = 0%). The findings indicated that acupuncture treatment significantly outperformed non-acupuncture treatment in enhancing cognitive function among IS patients [MD = 3.20, 95% CI (2.30–4.10), *p* < 0.00001, 2 studies, 120 subjects]. Furthermore, the heterogeneity analysis confirmed the reliability of the study results (Figure 9).

### 3.5 Evaluation of publication bias

**Figure 10:**
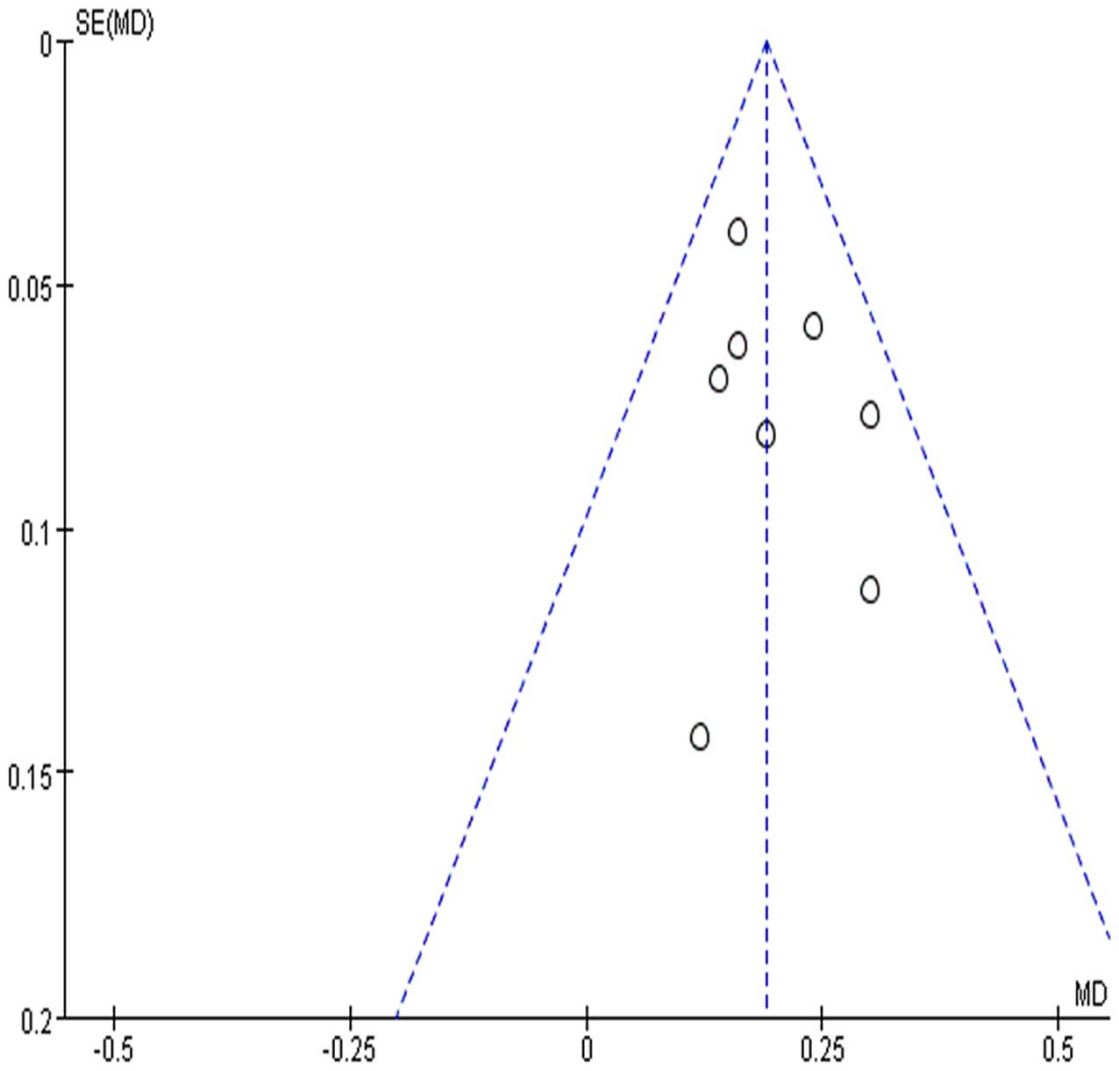
Funnel plot of acupuncture on NAA/Cr ratio in IS patients.

This study employed funnel plots and Begg’s test to assess publication bias. Analysis of the eight included studies reporting NAA/Cr ratios (18, 22, 27–32) showed that all results fell within the 95% confidence interval; however, funnel plots revealed slight asymmetry, suggesting possible mild publication bias (Figure 10). Concurrently, Begg’s tests for all outcome measures showed no significant publication bias for NAA/Cr ratio (*p* = 0.386), Cho/Cr ratio (*p* = 0.764), Lac/Cr ratio (*p* = 1.000), NIHSS score (*p* = 0.296), FMA score (*p* = 1.000), or MoCA score (*p* = 1.000). Details are presented in Table 2.

**Table 2:** Beeg test for publication bias of outcome measures reported in this study.

Outcome indicator	Begg’s test (*p*-value)	
NAA/Cr ratio	0.386	>0.05
Cho/Cr ratio	0.764	>0.05
Lac/Cr ratio	1.000	>0.05
NIHSS score	0.296	>0.05
FMA score	1.000	>0.05
MoCA score	1.000	>0.05

### 3.6 Quality of the evidence

**Figure 11:**
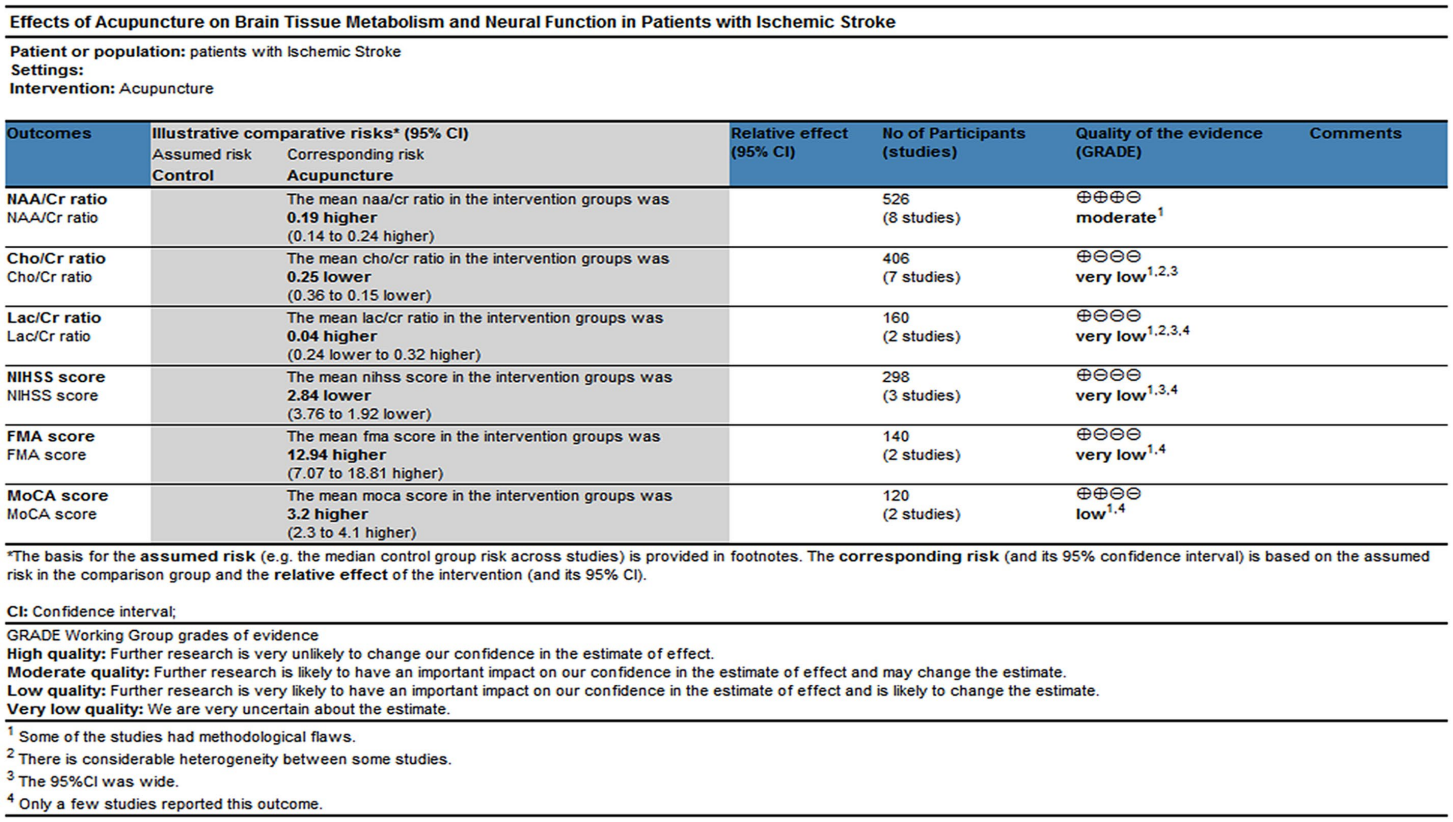
Level of evidence (GRADE).

This study assessed the quality of evidence utilizing the GRADE methodology and employed the online GRADEpro guideline development tool^2^. The GRADE criteria served as the foundation for evaluating the quality of the evidence. Downgrading was implemented when the number of low-risk assessments per study was fewer than the number of unclear or high-risk assessments. If this condition was met across all studies within each outcome indicator group, it was classified as ‘very serious.’ A heterogeneity exceeding 50% resulted in a one-level downgrade; similarly, if fewer than five studies reported on a given outcome indicator, a one-level downgrade was also applied. Consequently, the level of evidence for all measures varied from moderate to very low, as illustrated in Figure 11 for further details.

## 4 Discussion

### 4.1 Main findings

This systematic evaluation encompassed 9 items involving 602 IS patients and aimed to investigate the effects of acupuncture on brain tissue metabolism and neurological function in patients with focal cerebral infarction. The findings indicate that acupuncture outperforms conventional Western medicine, repetitive transcranial magnetic stimulation, and rehabilitation therapy in regulating brain metabolic levels (NAA/Cr and Cho/Cr ratios) at the lesion center in patients with ischemic stroke (IS). It also improves patients’ neurological function (NIHSS score), motor function (FMA score), and cognitive function (MoCA score). However, a significant degree of heterogeneity was observed in the integration of the Cho/Cr ratio within the lesion’s central area, with an *I*^2^ value of 70%. This study further analyzed potential influences on the heterogeneity of the results through subgroup analysis, sensitivity analysis, and forest plots. The subgroup analysis revealed that heterogeneity primarily stemmed from differences in intervention protocols among trial groups. Additionally, it may be associated with variations in study design (such as randomization methods, blinding, control group establishment, and allocation concealment) and sample characteristics.

### 4.2 Interpretation

After an IS, the structure and function of the human brain undergo numerous abnormal changes, which may subsequently lead to disorders in bodily functions (33). Therefore, promoting the recovery of brain structure and function is crucial for the rehabilitation of patients who have suffered an IS. Recent neuroimaging studies have demonstrated that acupoint stimulation can selectively activate functional areas of the cerebral cortex and positively influence neuroplasticity (34, 35). Studies have demonstrated that acupuncture can activate functional networks in damaged brain regions and promote plastic remodeling of brain structures in patients with IS, thereby significantly enhancing their neurological recovery (36–38). The mechanisms by which acupuncture mediates brain function remodeling in IS patients can be categorized into two primary aspects. Firstly, acupuncture can modulate key connections within brain networks. Following a cerebral infarction, the brain’s functional connectivity is disrupted, leading to the separation and reorganization of the default mode, sensorimotor, and salience networks. Research indicates that acupuncture can facilitate neurological recovery by enhancing functional connectivity within the sensorimotor network and its interactions with other brain regions (36, 39, 40). On the other hand, acupuncture may facilitate functional recovery by enhancing the synchronization and intensity of local neuronal activity (41, 42).

Gray matter cells in the brain undergo cell death and tissue necrosis following ischemic and hypoxic injuries, leading to varying degrees of alterations in gray matter volume and structure. These changes can facilitate the recovery of brain function by promoting the reconstruction of both gray matter volume and structure (43). Voxel-based morphometry (VBM) provides an objective, quantitative analysis of voxel size and signal intensity in the brain’s gray matter. It visually illustrates structural differences and dynamic changes in gray matter in patients with cerebral infarction, thereby providing a comprehensive view of gray matter volume changes (44). Acupuncture treatment significantly enhances the recovery of cerebral neural function in patients with hemiplegia following cerebral infarction. The underlying mechanism primarily involves remodeling of the gray matter structure within the extrapyramidal motor regulatory center and portions of the sensory cortex, facilitating functional compensation in the corresponding brain regions (45). Acupuncture treatment effectively promotes recovery of brain function by reconstructing the gray matter structure in functional areas of the brain closely related to human movement (46).

After ischemic injury to cerebral white matter, oligodendrocyte precursor cells can proliferate, differentiate under appropriate conditions, and migrate to the damaged area for repair. Diffusion Tensor Imaging (DTI) is highly sensitive to microstructural changes, enabling the observation of axon germination and myelin sheath proliferation. The corticospinal tract (CST) is the most vulnerable fiber bundle associated with motor dysfunction in patients with cerebral infarction, and its structural remodeling provides a theoretical basis for motor function rehabilitation following cerebral infarction. Using DTI, researchers found that the fractional anisotropy (FA) values of the affected corticospinal tracts at localized nodes in hemiplegic patients with IS increased following acupuncture treatment. This suggests that acupuncture may facilitate remodeling of the corticospinal tract by promoting axon sprouting and myelin sheath proliferation at specific nodes (47).

Ischemia of brain tissue leads to the swelling of nerve cells due to oxygen deprivation, accompanied by alterations in tissue structure. This change typically occurs within hours following a stroke and may initiate metabolic disturbances in the nerve cells, potentially exacerbating brain damage (48). Studies have confirmed that 1H-MRS-based metabolomics is a feasible and effective prognostic tool for assessing treatment efficacy in acute cerebral infarction. Common assessment metrics include brain tissue metabolites such as NAA, Cr, Cho, and Lac (49). NAA is a free amino acid predominantly found in the neurons of the adult brain and serves as a biochemical marker for evaluating neuronal viability in various neurological disorders, including cerebral ischemia. Estimates of NAA concentration provide insights into neuronal death during both early and late infarcts, and reductions in its levels can be utilized to assess the extent of neuronal loss and injury (50, 51). In the brain, Cho is associated with phosphoglycerol, phosphatidylcholine, and sphingolipids, and serves as a marker of cell membranes. The peak concentration of Cho is influenced by the levels of choline in membrane phospholipids and the availability of acetylcholine, a neurotransmitter. Pathophysiological processes can lead to sphingolipid breakdown, resulting in increased cell numbers and, subsequently, elevated Cho levels. Lac is the end product of anaerobic glucose metabolism and serves as a marker for cellular energy metabolism. Under normal conditions, the metabolism of brain neurons is predominantly aerobic, leading to very low lactate levels. However, when oxygen supply is insufficient, Lac levels rise, indicating ischemia in the brain’s vascular regions. The Cr value reflects the total concentration of creatine and phosphocreatine, remaining relatively constant and homogeneous across different metabolic conditions in the human brain. It is commonly used as a reference for other metabolites. Therefore, researchers often utilize the NAA/Cr, Cho/Cr, and Lac/Cr ratios to evaluate changes in brain tissue metabolism and the extent of brain damage following IS (49).

A decreased NAA/Cr ratio is closely associated with impaired neuronal or synaptic integrity and is commonly regarded as a metabolic marker of reduced or dysfunctional neuronal, axonal, and dendritic density (52, 53). One potential mechanism involves neuroinflammation driven by activated microglia: activated microglia directly cause neuronal injury and loss by releasing proinflammatory factors (54). Concurrently, elevated lactate concentrations within the lesion indicate macrophage infiltration and their high anaerobic glycolytic activity, while increased creatine levels further reflect hypermetabolic activity in microglia (55). The shared cellular lineage and response profiles of microglia and tissue macrophages suggest this represents a neuroinflammatory response (56). Furthermore, NAA serves as a precursor for neuroaspartic acid (NAAG), a neurotransmitter associated with synaptic plasticity. Thus, an increased NAA/Cr ratio not only indicates the rescue of damaged neurons but also signals a metabolic shift conducive to synaptic recovery and neural network reorganization, which are critical for restoring neural function. This systematic review found that acupuncture intervention significantly elevated the NAA/Cr ratio in the lesion area of ischemic stroke patients, suggesting its potential mechanism may lie in repairing or maintaining neuronal synaptic integrity by modulating neuroinflammatory responses and enhancing neural plasticity.

A reduced Cho/Cr ratio may reflect diminished membrane turnover or decreased inflammatory activity, while elevated levels of choline-containing compounds typically indicate increased phospholipid breakdown in cell membranes due to neuroinflammation and glial cell activation (e.g., astrocytosis and microglial proliferation) (52, 57). This meta-analysis demonstrates that acupuncture intervention significantly downregulates the Cho/Cr ratio in the lesion area of ischemic stroke patients, suggesting a potential mechanism involving downregulation of membrane turnover or inflammatory response. This finding is consistent with previous research conclusions (58).

This study integrates and analyzes recent research on the effects of acupuncture on brain tissue metabolism in patients with IS. The findings indicate that acupuncture can facilitate the remodeling of damaged brain nerve cells by regulating metabolic indices in brain tissue, thereby promoting the recovery of neurological function.

### 4.3 Publication bias and risk of bias

Funnel plots of NAA/Cr ratios, an outcome metric, exhibit slight asymmetry, indicating a potential risk of publication bias or other biases in trial inclusion. This may be attributed to the tendency for positive findings to be published more frequently, thereby inflating the effect size. Furthermore, the clinical competence of acupuncture practitioners varied across studies. Additionally, as all trials were sourced from a Chinese database, language and regional factors may also contribute to bias. Lastly, clinical heterogeneity, such as differences in intervention methods among trials, may represent another significant factor contributing to the observed asymmetry. To further assess publication bias, Begg’s tests for all outcome measures showed no significant evidence of bias.

An assessment of risk of bias in the included studies revealed significant methodological limitations (e.g., unclear randomization, inadequate allocation concealment, and lack of blinding), which may directly affect the final pooled effect estimates. Specifically, “some concern” or “high risk” ratings were prevalent in the domains of performance bias and detection bias, potentially leading to an overestimation of treatment effects. Furthermore, the invasive nature of acupuncture treatment makes blinding difficult to implement for participants, potentially allowing treatment effects to compound with placebo effects and overestimate outcomes. Therefore, implementing blinding in acupuncture research remains a current challenge.

### 4.4 Quality of evidence

In this study, the quality of evidence for all outcome indicators was assessed using the GRADE Pro guideline. It was determined that the quality of evidence for NAA/Cr ratios was moderate, while the quality of evidence for the outcome indicators Cho/Cr ratios, Lac/Cr ratios, NIHSS score, FMA score, and MoCA score ranged from low to very low. After thorough discussion, the review group concluded that the primary reasons for the decline in the level of evidence in this study were: (1) Methodological flaws present in some studies. (2) Significant heterogeneity among certain studies. (3) Wide 95% confidence intervals. (4) A limited number of studies report these outcomes. Therefore, although this study’s findings suggest that acupuncture may have beneficial effects on brain tissue metabolism and neurological function in patients with IS, further randomized controlled trials employing high-quality methodologies are necessary to strengthen the evidence base.

## 5 Strengths and limitations

This study represents the first systematic review of the existing literature investigating the effects of acupuncture treatment on brain tissue metabolism and neurological function in focal centers of IS patients. Additionally, this study offers a comprehensive evaluation of brain tissue metabolism and neurological function in IS patients using multiple outcome indicators, thereby providing substantial evidence for clinical practice.

This systematic literature review has several limitations. First, all nine included trials were conducted in China and published in Chinese, potentially limiting the generalizability of findings and introducing language/regional bias. This also confirms China’s central role in acupuncture stroke research and the growing trend toward integration with neuroimaging studies. Future high-quality trials involving international collaboration are needed to enhance the generalizability of research outcomes (59). Second, key methodological aspects such as randomization, allocation concealment, blinding, and follow-up were poorly described in the included studies, suggesting that errors in randomization methods may have contributed to some “positive” analytical outcomes. Third, there were variations in the acupuncture methods and the selection of acupuncture points among researchers, which may have led to substantial heterogeneity. Future studies should adhere to the Consolidated Standards of Reporting Trials (CONSORT) (60) and the Standards for Reporting Interventions in Clinical Trials of Acupuncture (STRICTA) (61) consensus to report details regarding needle insertion depth, subject responses, and therapist backgrounds. Finally, all included studies were single-center trials, which may limit the generalizability of the findings due to the specific characteristics of each institution’s population. Future research should enhance stability and generalizability by increasing the number of study centers and expanding the sample size of trial participants.

## 6 Conclusion

The results of the systematic evaluation and meta-analysis conducted in this study indicate that acupuncture treatment significantly enhances the regulation of brain tissue metabolites in the focal center of patients with cerebral infarction, and alleviates neurological deficits compared to conventional treatments, conventional rehabilitation therapy, and repetitive transcranial magnetic stimulation. Furthermore, acupuncture has been shown to benefit limb movement and cognitive function. While the findings show promise, they remain constrained by methodological limitations, necessitating cautious interpretation. Therefore, future research should focus on rigorously designed multicenter RCTs that employ standardized acupuncture protocols, blinded outcome assessments, and extended follow-up periods.

## Data Availability

The datasets presented in this study can be found in online repositories. The names of the repository/repositories and accession number(s) can be found in the article/Supplementary material.

## Footnotes

1
https://www.ncbi.nlm.nih.gov/mesh

2
https://gradepro.org/

## Glossary

RCTs: randomized controlled studies
WHO: the World Health Organization
IS: Ischemic stroke
MRS: magnetic resonance spectroscopy
NAA: nitrogen-acetylaspartate
Cr: creatine
Cho: choline
Lac: lactic acid
GRADE: the Grading of Recommendations, Assessment, Development, and Evaluation
MD: Mean differences
RR: relative risk
CI: confidence interval
FMA: Fugl–Meyer Assessment
rTMS: repetitive transcranial magnetic stimulation
MoCA: Montreal Cognitive Assessment
NIHSS: the National Institutes of Health Stroke Scale
VBM: Voxel-based morphometry
DTI: Diffusion Tensor Imaging
CST: Corticospinal tract
FA: fractional anisotropy
